# The Role of Humans in a Protracted Transition From Hunting-Gathering to Plant Domestication in the Fertile Crescent

**DOI:** 10.3389/fpls.2018.01287

**Published:** 2018-09-07

**Authors:** Terence A. Brown

**Affiliations:** School of Earth and Environmental Sciences, Manchester Interdisciplinary Biocentre, University of Manchester, Manchester, United Kingdom

**Keywords:** agriculture, domestication, emmer wheat, Fertile Crescent, human agency

Debate regarding the origins of agriculture in the Fertile Crescent tends to become polarized around two alternative models that view the transition from hunting-gathering to the cultivation of domesticated crops as either a relatively rapid and localized event or a protracted and dispersed process. The evidence supporting either model has been extensively reviewed (Brown et al., 2009; Abbo et al., 2010; Allaby, 2010), including a recent article (Abbo and Gopher, 2017) which emphasizes the longevity of the debate, which began with Darwin and has continued ever since, with the weight of opinion shifting regularly from one side to the other. The purpose of this Opinion is not to argue further the relative merits of the rapid-localized vs. protracted-dispersed models. Instead I focus on a single aspect of the two models, the part played by humans in the transition from foraging to growth of domesticated plants.

The rapid-localized model, by definition, envisages that the overall transition from gathering to plant domestication occurred during a few human lifetimes, with the genetic alleles that confer the basic domestication traits, which are rare in wild populations, rapidly becoming fixed in the crops. The pioneering experimental work of Hillman and Davies (1990) has shown that such a rapid fixation is possible even under an unconscious process, but requires efficient husbandry practices in order to apply the necessary selective pressures and to achieve reproductive isolation from wild plants. One implication of a rapid transition is therefore that the early cultivators possessed a clear understanding of the plant reproductive cycle, as well as a perception of which phenotypic traits would be beneficial for agriculture and an appreciation of how to exert the selective pressures needed to promote fixation of those traits. The extent of the human achievement represented by such a model is made even more impressive when we consider that the agricultural genesis involved not just one plant species, but a suite of crops that together provided a nutritionally-and agronomically-balanced package that would have been assembled via a set of conscious decisions (Abbo and Gopher, 2017).

In contrast, the protracted-dispersed model is looked on as involving much less, perhaps zero, direct and conscious human contribution in order to drive the process from gathering to domestication. Proponents of this model tend not to discuss the human dimension, but a constrained role for humans is implicit in those approaches that view agricultural origins as a coevolutionary mutualism between plants and humans (e.g., Rindos, 1984), and which contend that the processes leading to domestication of crop plants can be understood by study of non-human forms of “agriculture,” such as the ant-fungus mutualism (Schultz et al., 2005). Even when evolutionary approaches are based on the principles of human behavioral ecology (e.g., Gremillion et al., 2014a), the emphasis remains on the activities of human populations driven by ecological determinants (Zeder, 2015). Supporters of such approaches argue that their models do not ignore the potential role of intentional human agency in plant domestication (Gremillion et al., 2014b), but it is undeniable that evolutionary and ecological approaches to any problem are designed to place emphasis on the activities of populations rather than of individual organisms.

The role that humans played in the early development of agriculture is an emotive subject for many researchers. The notion that an analytic and intuitive “*La Pensée Savage*” (Levi-Strauss, 1962), with sophisticated floristic knowledge accumulated over the millennia, consciously assembled the suite of domesticated crops which subsequently drove the social and cultural changes that led to today's world systems is attractive both as an anthropological concept (Abbo et al., 2010; Abbo and Gopher, 2017), and as a reflection of popular perceptions of the prehistoric world (Ruddick, 2009). It is certainly a more alluring interpretation of the human past than the alternative, that our ancestors were unreasoning dumb partners in a process driven by ecological and evolutionary forces. But is it justifiable to criticize the protracted model, to the extent of suggesting that it cannot be correct, on the grounds that it assigns only a limited role to human agency? Or, to be more precise, is it correct to assume that a protracted process must by necessity equate to humans as dumb partners?

Bruce Smith and others (Smith, 2001; Jones and Brown, 2007) have described how, in a protracted model, the transition from gathering to cultivation of domesticated plants should be viewed as a series of incremental steps, each improving or making more secure the control exerted by humans over their plant food sources. Obtaining direct evidence of the nature of these steps is difficult, but possible events have been proposed based on ethnographic studies, with the underlying assumption that the practices of extant foraging populations reflect to some extent the activities of prehistoric hunter-gatherers (e.g., Harris, 2007; Rowley-Conwy and Layton, 2011). Based on these various studies, one is able to infer a series of possible steps that would lead from gathering of a wild grass to cultivation of a domesticated cereal crop (Figure 1). The model depicted in Figure 1 might or might not give an accurate representation of the process by which cultivation of domesticated cereals such as wheat or barley emerged in the Fertile Crescent, but this is immaterial. The key point is that if the transition was protracted then it is likely to have involved a punctuated series of steps of some description, each resulting in a small advance in plant management, rather than an unstructured and vague progression from gathering of wild plants to cultivation of a domesticated crop. The steps shown in Figure 1 might not be correct, but they are sufficiently representative of the likely incremental advances that did occur to be used to ask what role humans might have played during a protracted transition.

**Figure 1:**
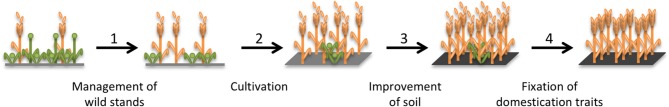
Schematic of a protracted model for the transition from gathering of a wild grass to cultivation of a domesticated cereal crop. The key steps are: (1) management of wild stands by removal of competing plants in order to increase the proliferation of the food sources; (2) initial cultivation of wild plants in artificial stands; (3) improvement of the soil; (4) fixation of domestication traits.

Let us therefore consider each of the steps in Figure 1 in turn and ask how they might have come about. First, the model envisages that gatherers manipulated the local environment to encourage the proliferation of their favored food sources, for example by removing competing plants or by supplementing wild stands with seeds of the favored species (Zvelebil, 1994). Of course, many animals manipulate their environments in ways beneficial to that individual animal, but the envisaged range management carried out by humans is more complex as the effects are not immediate, at least not for an annual cereal such as wheat or barley, where the increased density of plant growth occurs only after the existing plants complete their reproductive cycle. Step 1 therefore requires not only that humans understand the plant reproductive cycle, but also that they have the foresight to appreciate that actions performed today might only yield benefits in the following season, and that those actions might be counter-intuitive to immediate needs, as management of a stand of vegetation is worthless unless some of the food plants are left to complete their annual cycle, rather than all being gathered for immediate consumption.

The appreciation that the natural environment can be changed in a useful and consistent manner conceivably set the stage for step 2 in this particular protracted model, where the gathering of wild seeds is replaced, perhaps gradually, by the growth of artificial stands, which in northern Syria may have begun as early as the 10th millennium BC (Wilcox et al., 2008). One of the drivers leading to cultivation could have been the observation that spikelets or stray grains that escape processing and fall to the ground give rise in the following year to new plants. Or possibly the understanding of plant reproduction needed for range management was sufficient on its own to encourage humans to experiment with the artificial sowing of seeds in areas from which competing vegetation had been removed. In either case, there is a clear and important requirement for conscious human activity, planned to achieve a desired end that provides benefits that are anticipated before the experiment is carried out.

At some point during cultivation of the predomesticated crop we envisage that humans began to improve the soil of wild or cultivated stands. This could have involved tillage of soil, which is thought to have been practiced by Natufian communities during the 10th millennium BC or earlier (Barker, 2006, 126), or even relatively sophisticated techniques such as nitrogen supplementation, which Bogaard et al. (2013) suggest might date to the earliest farmers in southwest Asia. As with the previous steps in the sequence from foraging, soil improvement requires observation (e.g., plant growth is more productive on broken soil) followed by experiment (use of implements to prepare soil where plants are grown) and evaluation of the outcome: in other words, a typical example of hypothetico-deductive reasoning.

The final step in the protracted model involves fixation of the domestication traits. In the model depicted in Figure 1, this stage is completed relatively quickly and could even be interpreted as equivalent to the events occurring during a rapid transition, with the same implications for conscious human input. Other evidence suggests that the emergence of domestication traits such as nonshattering ears and seed size resulted from the application of relatively small selection pressures (Purugganan and Fuller, 2011; Fuller et al., 2014), which argues that the early farmers did not actively attempt to convert their predomesticated crop into a domesticated one. The latter proposal implies a lack of foresight and intuition that is, for some researchers, the key weakness of the protracted model, on the grounds that it is incompatible with the high degree of awareness that would be required to develop the sophisticated husbandry practices needed to maintain a healthy crop, whether predomesticated or domesticated. However, this argument places the horse before the cart: it implies that farmers invented husbandry practices in order to select for and achieve fixation of the domestication traits. The possibility that the development and refinement of these husbandry practices during a protracted process occurred not simply in order to achieve fixation of domestication traits, but more generally as a means of gradually improving the efficiency of crop management, does not in any way downplay the degree of human intuition and awareness that was needed to establish those practices.

I do not attempt to prove that a protracted origin of agriculture *must* have involved conscious human activity, but I do suggest that if we accept that a protracted origin involved a series of incremental steps, then we should also accept that conscious human activity is likely to have driven most or all of those steps. My hope is that acceptance of this point will enable the debate on agricultural origins to progress beyond the current suggestion that an implicit component of the protracted model is the assumption that during the Epipalaeolithic the hunter-gather communities of the Fertile Crescent were unable, or simply did not attempt, to make any conscious improvement to their subsistence strategies.

## Author contributions

The author confirms being the sole contributor of this work and approved it for publication.

### Conflict of interest statement

The author declares that the research was conducted in the absence of any commercial or financial relationships that could be construed as a potential conflict of interest.
